# Clinical strategies to manage adult glioblastoma patients without MGMT hypermethylation

**DOI:** 10.7150/jca.63595

**Published:** 2022-01-01

**Authors:** Delin Liu, Tianrui Yang, Wenbin Ma, Yu Wang

**Affiliations:** Department of Neurosurgery, Peking Union Medical College Hospital, Beijing, China.

**Keywords:** glioblastoma, MGMT, methylation, therapy, clinical trial

## Abstract

Glioblastoma (GBM) is a highly malignant brain tumor with a dismal prognosis. Standard therapy for GBM comprises surgical resection, followed by radiotherapy plus concomitant and adjuvant temozolomide (TMZ) therapy. The methylation status of the O6-methylguanine DNA methyltransferase (MGMT) promoter is one of the most essential predictive biomarkers for patients with GBM treated with TMZ. Patients with an unmethylated MGMT promoter (umMGMT), who comprise 60% of patients with GBM, present an even worse prognosis because of TMZ resistance. Radiotherapy with various fractionation, chemotherapy compensating for TMZ, targeted therapy against diverse oncogenic pathways, immunotherapy of vaccine or immune checkpoint inhibitor, and tumor treating fields have been studied in umMGMT GBM patients. However, most efforts have yielded negative results or merely minimal improvements. Therefore, effective patient subgroup selection concerning precision medicine has become the focus. By assigning different treatments to the corresponding patient subgroups, a better curative effect and subsequently prolonged survival can be achieved. In this review, we re-evaluate the value of standard TMZ therapy and summarize the new clinical strategies and attempts to treat patients with umMGMT, which yielded positive and negative results, to provide alternative treatment options and discuss future directions of umMGMT GBM treatment.

## 1. Introduction

Glioblastoma (GBM) is the most common and aggressive primary malignant brain tumor in adults, accounting for 54% of all adult gliomas 1. Despite optimized medical care, the median overall survival (OS) remains unsatisfactory. Standard therapy for newly diagnosed GBM includes surgical resection at maximum safety, followed by radiotherapy, as well as concomitant and adjuvant temozolomide (TMZ) therapy 2,3; however, the population-based 5-year survival rate is still less than 10% 4,5.

**Figure 1:**
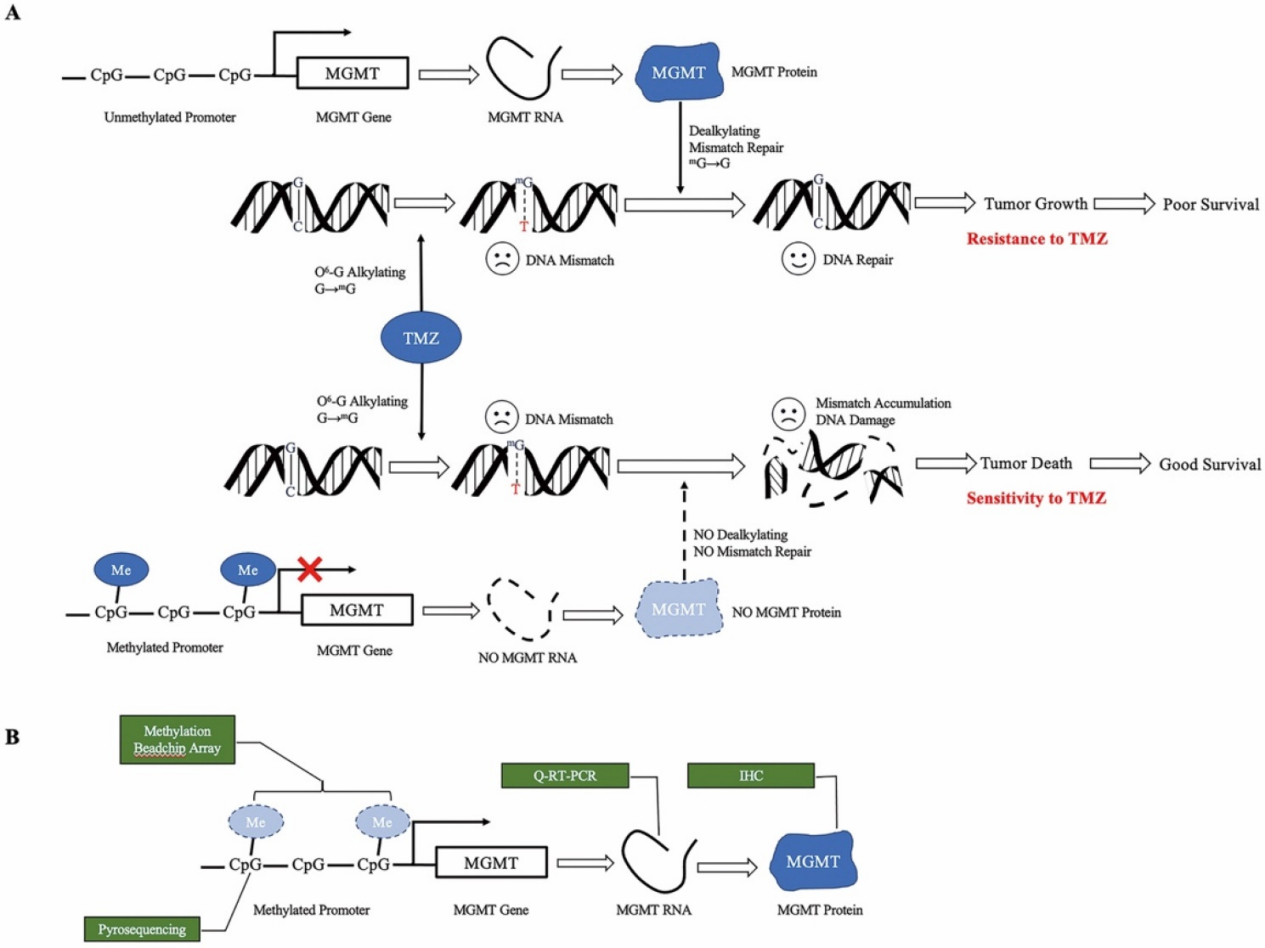
** Mechanism of MGMT promoter methylation. a.** Proposed role of MGMT promoter methylation as a predictive biomarker for TMZ. TMZ methylates the O6 position of guanine, leading to DNA mismatch. Unmethylated MGMT promotes TMZ resistance. When the MGMT promoter is unmethylated, transcription of the MGMT gene results in high MGMT protein expression, which is able to remove the alkylation adducts of mG and repair the mismatch, leading to tumor growth. When the MGMT promoter is methylated, silencing of transcription results in low level MGMT protein expression. Without dealkylation by MGMT protein, mismatch accumulates and causes DNA damage, leading to tumor death. **b.** Approaches to measure MGMT methylation status. Methylation BeadChip array analyzes the methylation spectrum of MGMT promoter. Pyrosequencing measures the average degree of methylation at the CpG site of MGMT promoter to determine the methylation status. Quantitative RT-PCR and immunochemistry analyze the level of MGMT RNA and protein, respectively. TMZ, temozolomide, MGMT O6-methylguanine DNA methyltransferase, mMe methyl, G guanine, mG O6-methylguanine, T thymine, Q-RT-PCR quantitative real-time PCR, IHC immunohistochemistry.

TMZ is an alkylating agent that methylates the guanine base of DNA, inducing futile mismatch repair 6-8. O6-methylguanine-DNA-methyltransferase (MGMT) removes the alkyl group, reversing the effect of TMZ. The expression of MGMT is silenced by MGMT promoter methylation 9-11. Therefore, the unmethylated status of the MGMT promoter is one of the most important causes of TMZ resistance, leading to a significantly more dismal survival rate 2,3,12-14 (Figure 1a). A meta-analysis pooled the survival from five phase III clinical trials, suggesting the OS and progression free survival (PFS) of umMGMT were 14.11 months and 4.99 months, respectively 15. Notably, MGMT is a reliable predictive biomarker for GBM in adults and the elderly; however, the value in children is less remarkable 16. In the newly released 2021 WHO classification of CNS tumors 17, glioblastoma lies in the adult diffuse glioma category, whereas pediatric gliomas are separately grouped with other important molecular markers such as H3K27-alteration and H3G34 mutation. Therefore, adult glioblastoma patients are mainly discussed in this review.

Great debate over the use of TMZ in umMGMT patients has existed since the discovery of MGMT as a biomarker. In this review, we summarize clinical studies that aimed to treat patients with umMGMT GBM and reported both positive and negative outcomes, and discuss current treatment strategies and future treatment directions for patients with umMGMT GBM.

## 2. Defining unmethylated MGMT

To date, the MGMT status has been measured at the protein level using immunohistochemistry (IHC), RNA level using quantitative real-time PCR (Q-RT-PCR), promoter methylation level using pyrosequencing, or as methylation profiling with the Illumina BeadChip array^18^ (Figure 1b).

The assessment of the MGMT status at the protein or RNA level was unable to predict survival. IHC has yielded inconsistent results 19,20, because of observer variability and poor reproducibility. Q-RT-PCR that measures RNA levels has yielded similar results in differentiating the predictive value 19. This limited discrimination is probably because of the mixed RNAs extracted from normal cells.

Pyrosequencing is currently considered the most reliable approach to quantitatively measure the methylation status 20,21. Methylation of the MGMT promoter is not a qualitative parameter with only two modes. Instead, quantitative measurement is needed. A retrospective study in Norway analyzed 48 GBM patients, and the cutoff value with the best prognosis was set to 7% methylation of MGMT using pyrosequencing 21. Another study in Italy set the cutoff similarly at 9% 22. Studies have also discovered a grey zone in between, which comprises approximately 10% of patients. Patients with hypermethylated and grey zone MGMT have better prognoses than those with umMGMT 23.

In recent years, studies have attempted to measure MGMT methylation in cell-free DNA (cfDNA) using methylation-specific PCR or pyrosequencing when the tumor tissue is not available. Although the results of pyrosequencing of cfDNA correlated with OS and PFS, both methods have low sensitivity and high negative predictive value 24. Additionally, researchers have attempted to evaluate the MGMT methylation status using imaging parameters, such as the apparent diffusion coefficient 25,26; however, these results have not been accepted to measure MGMT in clinical cases.

## 3. Strategies to treat umMGMT patients

### 3.1. Surgery

Surgical resection has been considered imperative for newly diagnosed GBM (nGBM) and possesses a certain value for recurrent GBM (rGBM). A greater extent of surgical resection of the contrast-enhancing tumor volume (CE-TV) is strongly associated with both prolonged OS and PFS 3,27-30. Gross total resection (GTR) is associated with even longer OS (31 months) and PFS (6 months) than subtotal resection (STR) (OS 15 months, PFS 4 months) in umMGMT patients. A retrospective study reported that the extent of resection (EOA) beyond 70% and residual tumor volume (RTV) below 1.5 cm^3^ is already of better prognosis for umMGMT patients 31. This result is stricter for mMGMT patients (98% and 1 cm^3^, respectively). However, a study by Sales et al. focusing on umMGMT patients suggested that complete resection of the CE-TV did not result in improved survival 32. Therefore, maximum safe resection, which avoids an aggressive surgical approach, is recommended instead of complete resection of important functional structures.

Laser Interstitial Thermal Therapy (LITT) is a new technique available as an alternative for deep, irresectable GBM patients, which utilizes the laser heat effect to destroy tumor cells with minimal invasion. LITT has been reported to annihilate deep-seated nGBM, rGBM, recurrent brain metastasis and radiation necrosis 33-35. A cohort of 20 nGBM patient has reached OS of 36.2 months and PFS of 3.5 months after LITT operation 36. For now, LITT works as a salvage treatment option only when open surgery is not applicable.

### 3.2. Standard TMZ radiochemotherapy

The phase III EORTC-NCIC trial indicated that radiotherapy (60 Gy/30) plus concomitant (75 mg/m^2^/d) and adjuvant (150-200 mg/m^2^ for 5 days each 28-day cycle) TMZ therapy was able to improve OS and PFS for nGBM patients compared with radiotherapy alone. This treatment, known as the Stupp's protocol, is now deemed the standard therapy for nGBM patients, especially for those with a methylated MGMT promoter. UmMGMT patients also benefit from radiotherapy plus TMZ, with a prolonged OS and PFS 12. The major problems of the standard therapy are acquired TMZ resistance especially for umMGMT patients and intolerance of adverse effects due to TMZ or radiation, such as nausea, neutropenia, brain edema and radiation necrosis. For elderly patients who are not able to tolerate the intensiveness of standard radiochemotherapy, adjusted treatment plans are available. Hypofractionated radiation (40 Gy/15) plus TMZ was effective in a phase III clinical trial 37. If even hypofractionated radiochemotherapy is intolerable, umMGMT GBM patients should choose radiotherapy alone over TMZ alone 13,14. The effectiveness of radiotherapy will be discussed in detail in section 3.3.

### 3.3. Optimizing radiotherapy

Efforts have been made to investigate whether an adjusted radiotherapy method could further improve survival. A retrospective study applied dose-escalated radiotherapy (70 Gy/35) plus standard concomitant and adjuvant TMZ and proved that it significantly prolonged the OS and PFS of umMGMT patients. The median OS increased from 8 months to 14 months, and PFS increased from 5 months to 9 months. This study was the first to moderately escalate radiation specifically in patients with umMGMT and was confirmed effective 38. A recent meta-analysis reported that dose-escalated radiotherapy exceeded the effect of standard radiotherapy alone 39. However, when combined with temozolomide, the superiority was insignificant.

Dose-escalated radiotherapy was also co-registered with positron emission tomography (PET) to better delineate the target volume. A prospective phase II clinical trial utilized 3,4-dihydroxy-6-[18F]-fluoro-L-phenylalanine (^18^F-DOPA) PET guided dose-escalated radiotherapy(51~60~76 Gy/30)^40^. In umMGMT patients, 6-months PFS (PFS-6) reached 79.5% (primary end-point set at 75%) and PFS was 8.7 months compared to the historical control of 6.6 months.

Among all nGBM patients, elderly patients appear to rely more on radiotherapy, since elderly patients are likely to be more sensitive to the toxicity of intensive chemotherapy. The phase III NOA-08 trial suggested that the event-free survival (EFS) was longer in elderly umMGMT patients treated with radiotherapy alone than in patients treated with TMZ alone (100 mg/m^2^) 14,41. And the OS of radiotherapy alone was no worse than TMZ alone. A meta-analysis suggested that radiotherapy alone was associated with a longer OS. Moreover, TMZ alone was associated with increased side effects in patients with umMGMT.

One of the purposes of hypofractionated radiotherapy is to reduce the number of hospital visits and avoid drop-out due to deterioration or disease progression. A phase III trial, the Nordic trial, compared TMZ alone, hypofractionated radiotherapy (34 Gy/10) and standard radiotherapy 13. Patients generally responded better to TMZ alone and hypofractionated radiotherapy. The treatment effect of hypofractionated radiotherapy was independent of the MGMT methylation status. Therefore, for patients with umMGMT, hypofractionated radiotherapy might be considered prior to TMZ treatment. Another phase III trial used hypofractionated radiation (40 Gy/15) plus concomitant and adjuvant TMZ 37. Hypofractionated radiotherapy plus TMZ was proven to be more effective than radiotherapy alone. The OS was 10.0 months compared with 7.9 months in umMGMT patients. This study illustrated that the addition of TMZ to radiation is, although less effective, still beneficial for elderly patients, even with a reduced overall radiation dose.

In summary, for adult umMGMT patients, radiotherapy plus concomitant and adjuvant TMZ was still more effective than radiotherapy alone. For patients older than 65 years of age, hypofractionated radiotherapy plus TMZ is an available choice. If one of the schedules must be eliminated from the plan, umMGMT patients should choose radiotherapy alone first, while mMGMT patients should consider otherwise.

### 3.4. Promising clinical trials

Despite all these improvements, the prognosis of umMGMT patients remains poor. Therefore, investigators have paved the way for clinical trials of novel or traditional chemotherapies and other therapies that could substitute or compensate for TMZ. However, the results were generally unsatisfying. Only a few treatments resulted in slight improvements. A list of clinical trials with both positive and negative results is summarized in Table 1.

#### 3.4.1 Tumor Treating Fields

Tumor treating fields (TTF) is an external device that can eliminate tumors regardless of the MGMT methylation status. TTF utilize an electric field to interfere with the mitosis of tumor cells and thus inhibit tumor progression. In a randomized phase III clinical trial, EF-14, including 695 nGBM patients 42-44, TTF plus TMZ were administered after radiotherapy. The addition of TTF was associated with prolonged OS and PFS for both mMGMT and umMGMT subgroups. Among the 304 umMGMT patients, OS was prolonged from 14.7 months to 16.9 months. Quality of life was not affected. A pilot study reported the initial result of concurrent radiotherapy plus TTF, which is supposed to induce synergistic effect 45. Toxicity was tolerable in this initial report.

TTF appear to be a promising treatment method apart from the limitations of chemotherapy. It has been officially included in National Comprehensive Cancer Network (NCCN) guideline of CNS tumors. TTF is independent of known molecular biomarkers of GBM, and can combine swiftly with standard therapy. The hindrance of TTF is that its effectiveness relies heavily on the put-on time per day, which cannot be reached in some patients due to intolerable skin reaction or poor patient compliance.

#### 3.4.2 Chemotherapy

Recently a phase II clinical trial recruited 47 nGBM patients with 31 umMGMT and applied the concomitant and adjuvant combination of temozolomide, vincristine and interferon 46. A 2-year overall survival was 40.7% for the entire cohort, exceeding the historical control. PFS reached 11.0 months irrespective of MGMT methylation status. This study revealed a possible combination of chemotherapy drugs in addition to temozolomide. However, the increased adverse effect may limit the use of this study plan.

Dianhydrogalactitol (VAL-083) is another kind of DNA-alkylating agent independent of MGMT repair and temozolomide pathway. In the halfway report of this phase II clinical trial targeting umMGMT nGBM, 10 patients remained progression free, while another 12 patients progressed with PFS of 9.9 months 47. It turns out that VAL-083 may be promising for umMGMT patients in substitute of temozolomide. However, this conclusion awaits further clinical results.

Paclitaxel poliglumex (PPX) is a polymer agent derived from the traditional chemotherapy agent paclitaxel. In phase II clinical trial (BrUOG 244) 48, PPX (10 mg/m^2^) was administered during the radiotherapy session on the first day of each week to enhance tumor sensitivity to radiotherapy. Adjuvant TMZ therapy was commenced after radiotherapy. However, this study showed no clinical benefit for umMGMT patients.

#### 3.4.3 Integrin inhibitors

The significant efforts lie in targeted therapy. However, the study of cilengitide, an integrin inhibitor, has reached a dead end. Integrins are important proteins that mediate angiogenesis in nGBM and cell apoptosis. A previous phase I/II study of the CORE trial revealed that both standard and intensive cilengitide treatments were able to prolong the OS and PFS of umMGMT patients. Further analysis also showed that high αvβ3 integrin expression in umMGMT nGBM was associated with prolonged PFS independent of the treatment type 49. Nevertheless, in the subsequent phase III CENTRIC EORTC 26071-22072 study that only included mMGMT patients, cilengitide (2000 mg, twice a week) plus radiochemotherapy failed, with no survival benefit. Cilengitide has thereafter not been further studied as a treatment for GBM.

Another phase II trial specifically targeting umMGMT patients added procarbazine to cilengitide 50. Procarbazine is a DNA alkylating agent that has been shown to inhibit MGMT independent of TMZ in preclinical trials. Concomitant and adjuvant TMZ was replaced with TMZ (60 mg/m^2^) plus procarbazine (50-100 mg) during and after radiotherapy. However, this combination of targeted treatments did not produce a positive outcome.

#### 3.4.4 Anti-angiogenesis therapy

Another novel medication that has been extensively studied was bevacizumab, which resulted in prolonged PFS but not OS 51. Bevacizumab is an antibody against vascular endothelial growth factor (VEGF). In the study published by Gilbert et al., bevacizumab improved PFS from 5.4 months to 9.8 months in umMGMT patients. Another phase III trial published by Chinot et al. 52 documented similar results that only PFS was improved. And more adverse events were associated with bevacizumab than with placebo. A phase II trial ARTE targeted elderly patients. Bevacizumab and hypofractionated radiotherapy (40 Gy/15) was compared with radiotherapy alone, showing prolonged PFS but no OS benefit in umMGMT patients 53.

The combination of bevacizumab and other medications did not improve OS either. In the phase II GLARIUS trial, TMZ was substituted by bevacizumab plus irinotecan 54. The experimental arm showed an increase in the PFS-6 (from 42.6% to 79.3%) and PFS (from 5.99 to 9.7 months), but not OS. In another phase II trial specifically targeting umMGMT patients 55, erlotinib plus bevacizumab therapy were commenced after radiotherapy plus concomitant TMZ therapy. This adjuvant therapy was well tolerated but did not improve PFS or OS.

Although studies of bevacizumab have all shown improvement in PFS but not OS, bevacizumab is well effective in reducing brain edema and improving symptoms, as a substitute of glucocorticoids, but without the extensive long-term adverse effects. It has also shown potential benefit to the immune microenvironment of GBM 56. Therefore, bevacizumab is still an actively used agent in clinical practice.

#### 3.4.5 PKC/PI3K inhibitors

Approaches to intervene in other oncogenic pathways have also been researched. In a phase II clinical trial 57, enzastaurin, a PKC inhibitor, was administered explicitly to umMGMT patients before, during and after radiotherapy. In this single-arm study, while the dose was safely tolerated, the primary endpoint PFS-6 was unfortunately not reached. For umMGMT patients who underwent partial or complete resection, the OS was 15.4 and 18.9 months, respectively. Nonetheless, PFS-6 is no longer used as the primary objective in many trials because of its limitations. Therefore, although failing to meet its primary endpoint, enzastaurin should be considered a promising agent for umMGMT patients.

#### 3.4.6 mTOR inhibitor

In the phase II clinical trial EORTC 26082 58, radiotherapy plus concomitant and maintenance temsirolimus, an mTOR inhibitor, was administered at a dose of 25 mg per week. Neither OS nor PFS was significantly improved for umMGMT patients. A further subgroup analysis showed that a particular group of mTOR (Ser2448)-positive patients may benefit from radiotherapy plus temsirolimus treatment. Further clinical evidence is needed to confirm this finding.

#### 3.4.7 Protease inhibitors

In addition to traditional chemotherapy, protease inhibitors have also been considered. However, a phase II clinical trial declared that the umMGMT subgroup had a relatively poor prognosis after treatment with standard radiochemotherapy plus bortezomib (1.3 mg/m^2^ on days 1, 4, 8, and 11 of the 28-day cycle) 59. Although this trial recruited only 23 patients (only 13 umMGMT patients), the overtly poor outcome was unlikely to be reversed with the inclusion of more patients.

Veliparib is a poly ADP-ribose polymerase (PARP) inhibitor that kills tumor cells and sensitizes radiotherapy in preclinical studies 60. The phase II clinical trial VERTU, particularly targeting umMGMT patients, added concomitant and adjuvant veliparib to standard radiochemotherapy 61. PFS-6 achieved 46% over 31% in experiment arm and control, respectively. However, OS was not prolonged (12.7 vs. 12.8 months).

#### 3.4.8 Anti-EGFR antibodies

Likewise, nimotuzumab, an EGFR antibody, was a beneficial treatment with predictive biomarkers from the Akt and mTORC signalling pathways. In an open-label phase III trial 62, nimotuzumab was added to standard TMZ therapy. For patients with umMGMT and EGFR amplification, PFS increased from 5.8 to 8.3 months, while OS from 15.5 to 19.5 months. A further subgroup analysis was conducted 63. Akt and mTORC, which serve as downstream effectors of EGFR inhibition, have been defined as positive predictive biomarkers for nimotuzumab treatment. However, most patients do not experience this potential benefit, and for those included in the study, the outcome was not as promising as that observed in mMGMT patients.

#### 3.4.9 Immunotherapy

DCVax-L was a phase III clinical trial that utilized the autologous tumor lysate-pulsed dendritic cell (DC) vaccine to treat nGBM patients^64^. DCVax-L was administered after surgery and radiotherapy, with a dose of 2.5 million DC intradermally. For the umMGMT group, OS was 19.8 months from surgery. The limitation of this study is that patients with symptoms of early progression were excluded from the study cohort. Therefore, selection bias may have contributed to the good OS outcome.

Recently, the checkpoint inhibitor nivolumab was also reported to have failed in a phase III trial in umMGMT (Checkmate 498) and mMGMT (Checkmate 548) patients. Nivolumab was added as an adjuvant to standard radiochemotherapy. The primary endpoint, OS, was not reached by the time of the announcement, although toxicity was well tolerated. On the other hand, the use of a neoadjuvant PD-1 inhibitor in GBM may be promising. In a phase II clinical trial analyzing neoadjuvant PD-1 in 27 rGBM and 3 nGBM patients, increased immune activity was detected 65. Two of the three nGBM patients, both of whom were mMGMT, survived for over 28 and 33 months, respectively. The efficacy and effectiveness of a neoadjuvant PD-1 inhibitor await future investigation.

#### 3.4.10 Virus-related treatment

According to previous studies, patients with GBM are often diagnosed with cytomegalovirus infection, potentially a treatment target. In the phase III clinical trial ASPECT, adenovirus-mediated gene therapy called sitimagene ceradenovec was commenced to treat nGBM patients 66. This genetically altered adenovirus carrying a prodrug converting enzyme for ganciclovir was directly injected into the tumor cavity, followed by intravenous ganciclovir, radiotherapy with or without TMZ. In the umMGMT subgroup, patients had prolonged time to death or re-intervention, regardless of TMZ use. Patients with umMGMT experienced a greater benefit than mMGMT patients.

A retrospective study of GBM patients receiving valganciclovir in addition to standard radiochemotherapy has yielded positive results for umMGMT patients. Valganciclovir exerts an anti-cytomegalovirus effect and has significantly improved the OS for umMGMT patients from 11.6 months to 21.1 months. Although this retrospective study only included some eligible patients for the MGMT methylation analysis, valganciclovir represents a promising treatment option and future direction.

The first oncolytic virus therapy in GBM has been approved by the Japan Ministry of Health, Labour and Welfare in 2021. Teserpaturev (Delytact/G47) is a herpes simplex virus 1 (HSV-1) oncolytic virus that has been genetically modified to selectively replicate in cancer cells 67. The correlated phase II clinical trial outcome has not been published yet.

### 3.5. Recurrent glioblastoma

Unlike nGBM, a well-defined standard therapy is not available for rGBM patients. Strategies, such as a second resection, re-irradiation, bevacizumab, lomustine and TMZ rechallenge, are generally considered beneficial.

A second resection should only be performed when a survival benefit will be obtained. A retrospective study has found that rGBM patients who underwent GTR and STR of the tumor experienced longer OS than patients with biopsy or without surgery 68. For umMGMT rGBM patients, the superiority of GTR over STR was also noted. Another study, however, suggested a more conservative attitude toward a second resection. In the post hoc analysis of the cohort in the DIRECTOR trial, patients experienced prolonged survival only if GTR of the advancing tumor can still be achieved at the first recurrence 69. Therefore, the choice of a second resection at recurrence should be carefully evaluated for individuals considering the personal needs, functional status, tumor size and location, preferably only if GTR is possible.

At the time of relapse, the MGMT promoter methylation status is changed bidirectionally in a range of 8-37% GBM patients due to selective pressure and intratumor heterogeneity 70. More patients lost MGMT methylation than gained. This causes complications and opportunities for TMZ rechallenge. In both intensive or low dose TMZ rechallenge 71,72, mMGMT patients showed significantly longer PFS than umMGMT patients. The phase II GICNO trial 73 suggested that TMZ (75 mg/m^2^/d) was able to increase the PFS-6 for rGBM patients regardless of MGMT status. Though other studies still regarded MGMT methylation as a predictive biomarker of rGBM.

In addition to TMZ rechallenge, antiangiogenic therapy is one of the most commonly used therapies for treating rGBM patients. For umMGMT patients, one promising treatment combines onartuzumab, an anti-MET antibody, with bevacizumab 74. The phase II clinical trial evaluated onartuzumab (15 mg/kg) plus bevacizumab (15 mg/kg) and showed a negative effect on the overall cohort, especially mMGMT patients. However, in the umMGMT subgroup, the results indicated prolonged survival and slower progression, suggesting that onartuzumab plus bevacizumab might be a promising treatment for umMGMT patients. Further clinical evidence targeting umMGMT patients is needed.

### 3.6. Future directions

Ongoing phase II or phase III clinical trials either specifically designed for patients with umMGMT or a cohort of umMGMT patients are listed in Table 2, including studies registered at clinicaltrials.gov, clinicaltrialsregister.eu, anzctr.org.au and chictr.org.cn. Two phase III trials were identified. Both of them were related to immune therapy. Several molecules, including immune checkpoint inhibitors, dendritic cells, protease inhibitors, PARP inhibitors, and mTOR inhibitors, are being investigated.

In the future, precision medicine may lead to higher therapeutic effectiveness. In a phase I/Ib clinical trial published in Nature in 2019, a personalized neoantigen vaccine was shown to trigger the immune response mediated by neoantigen-specific T cells in umMGMT GBM patients 75. A neoepitope-related peptide was designed specifically for each patient. This vaccine was able to trigger the T cell-mediated immune response and alter the immune environment of GBM. Further studies are needed to verify its effectiveness.

Another study design was published based on the idea of precision medicine. The phase I/II NOA-20 trial intended to match patients to the molecular subgroups 76. After the molecular analysis, the patients who possessed an ALK fusion, CDK4/6 amplification, mTOR phosphorylation, MDM2 amplification or SHH amplification were assigned to receive different targeted medications separately. Patients without those markers were randomized to use TMZ, asinercept (APG101) or the checkpoint inhibitor. This study design was based on sophisticated bioinformatics analyses and was believed to be more efficient for clinical trials.

## 4. Conclusions

The umMGMT status is a lynchpin factor that leads to TMZ resistance in GBM patients. However, only 40% of GBM patients carry hypermethylated MGMT, leaving 60% of patients with minimal benefits. In addition to surgical resection and standard TMZ radiochemotherapy, bevacizumab plus irinotecan, enzastaurin plus TMZ and TTF also benefit umMGMT patients to a certain extent. For elderly patients with umMGMT, hypofractionated radiotherapy plus TMZ is preferred when tolerable. If not, standard or hypofractionated radiotherapy alone should be considered superior to TMZ alone. For umMGMT GBM patients, recent clinical trials have failed to yield promising outcomes. Researchers have been studying the ways to reverse TMZ resistance or to overpass MGMT pathways. The success of TTF was an example of therapy or even external device with novel anti-tumor mechanisms. Because of the high inter- and intra-tumor heterogeneity and dynamic transformation of MGMT methylation status, precision medicine is currently of particular therapeutic value.

## Figures and Tables

**Table 1:** Outcomes of clinical trials for newly diagnosed GBM patients with unmethylated MGMT

Trial	Phase	Year	Experiment arm	Control	Mechanism	Benefit	Patient subgroup
EORTC-NCIC	3	2005, 2009	RT+ TMZ	RT		OS, PFS	
NOA-08	3	2012, 2020	RT	CRT		OS, EFS	Elderly
EORTC-26062-22061	3	2017	HF-RT(40Gy/15) +TMZ	HF-RT	RT	OS	Elderly
Nordic	3	2012	HF-RT(34Gy/10)	RT or TMZ alone	RT	OS	Elderly
NCT01991977	2	2021	^18^F-DOPA-PET+CRT	historical	PET+RT	PFS	
NCT00509821	2	2013	ENZ+RT	single arm	PKC	OS	
ASPECT	3	2013	Adenovirus +RT (+TMZ)	RT (+TMZ)	Gene therapy	PFS	
CORE	1/2	2015 2016	CIL+CRT	CRT	Integrin	OS, PFS	αvβ3 integrin
GLARIUS	2	2016 2018	BEV+IRI+RT	CRT	VEGF, topoisomerase	PFS6, PFS	
ARTE	2	2018	BEV+HF- RT(40Gy/15)	HF-RT	VEGF, RT	PFS	
EF-14	3	2017	TTF+CRT	CRT	TTF	OS, PFS	
retrospective study	-	2020	valganciclovir +CRT	CRT	anti CMV	OS, PFS	
EORTC 26082	2	2016	TEM+RT	CRT	PI3K	insignificant	mTORser2448
OSAG 101-BSA-05	3	2015	NIM+CRT	CRT	EGFR	OS	Akt, mTORC
ExCentric	2	2016	CIL+PRO+CRT	single arm	Integrin, DNA-alkylating	insignificant	
NCT00720356	2	2016	ERL+BEV+CRT	single arm	EGFR, VEGF	insignificant	
BrUOG 244	2	2018	PPX+RT	CRT	Paclitaxel	insignificant	
NCT00998010	2	2018	BOR+CRT	single arm	Protease inhibitor	insignificant	
VERTU	2	2021	veliparib+CRT	CRT	Protease inhibitor	PFS	
CHECKMATE 498	3	2019	NIV+RT	N/A	PD-1 inhibitor	insignificant	

RT, radiotherapy; TMZ, temozolomide; CRT, standard chemoradiotherapy; HF, hypofractionated; OS, overall survival; PFS, progression free survival; EFS, event free survival; CIL, cilengitide; BEV, bevacizumab; TTF, tumor treating field; CMV, cytomegalovirus;TEM, temsirolimus; NIM, nimotuzumab; ENZ, enzastaurin; PRO, procarbazine; ERL, erlotinib; PPX, paclitaxel poliglumex; BOR, bortezomib.

**Table 2:** Ongoing phase II/III clinical trials specifically for umMGMT patients or with umMGMT subgroup

	Experiment arm	Mechanism	Phase	Trial
1	Disulfiram+Copper Gluconate+CRT	Chemotherapy	2	NCT03363659
2	Nivolumab+Ipilimumab+ short-course RT	PD-1 inhibitor CTLA-4 inhibitor	2	NCT03367715
3	Nivolumab+Ipilimumab+RT	PD-1 inhibitor CTLA-4 inhibitor	2/3	NCT03367715 NCT04396860
4	CMV pp65 DC vaccine +CRT	Immune vaccine	2	I-ATTAC NCT03927222
5	CMV pp65 DC vaccine+ varlilumab+CRT	Immune vaccine anti-CD27	2	NCT03688178
6	Paxalisib+CRT	PI3K/mTOR inhibitor	2	NCT03522298
7	Dianhydrogalactitol+CRT	Chemotherapy	2	NCT02717962 NCT03050736
8	Olaptesed Pegol+RT	CXCL-12 inhibitor	1/2	GLORIA NCT04121455
9	Bortezomib+RT+TMZ	Protease inhibitor	1/2	BORTEM-17 NCT03643549
10	Temferon+RT	Lentivirus CD34+enriched HSPC	1/2	TEM-GBM NCT03866109
11	Pembrolizumab+CRT Pembrolizumab+HSPPC-96+CRT	PD-1 inhibitor Immune vaccine	2	NCT03018288
12	INO-5401+INO-9012+cemiplima+CRT	DNA plasmid PD-1 inhibitor	1/2	NCT03491683
13	APG101, Alectinib, Idasanutlin, Atezolizumab, Vismodegib, Palbociclib, Temsirolimus	Targeted therapy	1/2	N²M² (NOA-20) NCT03158389
14	Durvalumab +RT	PD-L1 inhibitor	2	NCT02336165
15	Pamiparib+RT, Pamiparib+CRT	PARP 1/2 inhibitor	1/2	NCT03150862
16	Sunitinib+CRT	TKI	2	NCT02928575
17	Chlorpromazine+CRT	dopamine receptor D2 antagonist	2	NCT04224441
18	Selinexor+RT	selective inhibitor of nuclear export	2	NCT04421378
19	Apatinib+TMZ	TKI	2	ChiCTR1900020561
20	Anlotinib+CRT	TKI	2	NCT04725214

RT, radiotherapy; CRT, standard chemoradiotherapy; TMZ, temozolomide; HSPC, Hydrogenated Soybean Phospholipids; TKI, tyrosine kinase inhibitor.
